# A Study of the Effect of the Levonorgestrel Intrauterine System on the Management of Abnormal Uterine Bleeding

**DOI:** 10.7759/cureus.96235

**Published:** 2025-11-06

**Authors:** Soumya S Patil, Vidya Jadhav, Nidhi Pathak

**Affiliations:** 1 Obstetrics and Gynaecology, Bharati Vidyapeeth (Deemed to be University) Medical College and Hospital, Sangli, IND

**Keywords:** abnormal uterine bleeding (aub), conservative approach, endometrial hyperplasia, heavy menstrual bleeding (hmb), levonorgestrel intrauterine system, menstrual blood loss reduction, patient satisfaction, uterine adenomyosis, uterus-sparing therapy

## Abstract

Introduction

Abnormal uterine bleeding (AUB) significantly impacts women’s quality of life. The levonorgestrel-releasing intrauterine system (LNG IUS 52 mg) is a uterus-sparing and effective treatment option.

Methods

This retrospective observational study included 27 women with AUB managed on an outpatient basis at a tertiary care centre. Data were collected from hospital records using a predefined proforma, Pictorial Blood Loss Assessment Chart (PBAC), and the SAMANTA questionnaire. Outcomes included haemoglobin change, bleeding pattern improvement, patient satisfaction, and complications. Validated tools and a minimum six-month follow-up strengthened reliability.

Results

The mean age of participants was 40.3 years. The mean haemoglobin level improved significantly from 9.4 g/dL to 11.6 g/dL (p < 0.0001). PBAC scores, including subscores for pad saturation and clot passage declined significantly after LNG-IUS insertion. The mean SAMANTA score, reflecting menstrual bleeding-related quality of life, reduced from 5.81 ± 2.97 to 0.52 ± 0.98 (p < 0.0001). Amenorrhea was achieved in 10 of 27 women (37%), and 25 (93%) reported treatment satisfaction. One patient (4%) experienced spontaneous device expulsion, and two patients (7%) discontinued use, one due to persistent bleeding and another due to weight gain. No participants reported mood or libido changes. The proportion of women with bleeding lasting more than seven days decreased from 74% to 0%, and none reported night-time staining or activity avoidance following insertion.

Conclusion

The levonorgestrel intrauterine system (LNG-IUS 52 mg) significantly reduced menstrual blood loss and improved haemoglobin and quality-of-life parameters in women with AUB. It was well tolerated, demonstrated a high continuation rate, and represents an effective uterus-sparing alternative to hysterectomy.

## Introduction

Abnormal uterine bleeding (AUB) significantly impacts women's quality of life, necessitating effective management strategies. Heavy menstrual bleeding (HMB) is one of the most common reasons for gynaecological consultations in both primary and secondary care. About 1 in 20 women aged between 30 and 49 years consult their general practitioner each year for heavy periods, and menstrual disorders account for approximately 12% of all gynaecology referrals [1]. In India, approximately 3.2-3.6% of women aged 15-49 have undergone hysterectomy, with rural areas showing higher rates (3.4-3.6%) compared to urban settings (2.5-2.7%) [2,3]. In Maharashtra, the overall rate is around 2.6%, though districts like Beed have reported alarmingly high figures (~36%), particularly among sugarcane workers [4]. In the Sangli-Sangola-Solapur region, hysterectomy has even become normalised among female agricultural labourers, driven by heavy physical work, menstrual concerns, and limited access to conservative care [5]. In such contexts, counselling patients about uterus-preserving options can be particularly challenging. Many women and families view hysterectomy as a socially accepted and quick solution to menstrual issues, making it difficult to introduce conservative therapies such as the levonorgestrel intrauterine system (LNG-IUS). This high burden underscores the need for effective, non-surgical treatment strategies.

The LNG-IUS has gained widespread use globally and is recommended as a first-line therapy for AUB. Recent systematic reviews have reinforced its role in clinical guidelines as a conservative yet effective treatment option [6].

Further supporting these findings, a 2009 systematic review concluded that the LNG-IUS not only improved quality of life but also reduced menstrual blood loss more effectively than standard medical therapies. Notably, this review found no significant differences in outcomes when comparing the LNG-IUS to endometrial ablation or hysterectomy, suggesting that the LNG-IUS offers a less invasive yet equally effective alternative to surgical interventions for AUB [7].

In a 2023 study focusing on patients with various underlying pathologies causing AUB, the LNG-IUS demonstrated an overall effectiveness rate of 82% in reducing menstrual bleeding. The efficacy was particularly pronounced in patients with endometrial hyperplasia and adenomyosis, with effectiveness rates of 95.5% and 88.7%, respectively. However, the effectiveness was comparatively lower in patients with leiomyoma, indicating that the underlying cause of AUB may influence the therapeutic success of the LNG-IUS [8]. We aimed to evaluate the clinical outcomes and patient-reported satisfaction following LNG-IUS insertion in women with AUB.

## Materials and methods

This was a single-institution retrospective observational study with a prospective follow-up component, conducted in the Department of Obstetrics and Gynaecology at Bharati Vidyapeeth (Deemed to be University) Medical College and Hospital, Sangli, Maharashtra, India. The study included 27 women diagnosed with abnormal uterine bleeding (AUB) who had undergone LNG-IUS (levonorgestrel intrauterine system 52 mg) insertion between January 2022 and November 2024. Institutional Ethics Committee (IEC) approval was obtained prior to data collection. As this was a retrospective study using hospital record data, informed consent was waived by the ethics committee. Patient confidentiality was strictly maintained, and all identifiable information was anonymised prior to analysis.

All reproductive-aged and perimenopausal women with AUB who had received LNG-IUS insertion and had a minimum of six months of follow-up data were included. Patients with fibroids distorting the uterine cavity, suspected or confirmed malignancy, unexplained vaginal bleeding, active pelvic infection, or congenital uterine anomalies were excluded. Data were extracted from medical records using a predefined proforma.

Baseline demographic data including age, parity, body mass index (BMI), and comorbidities were collected. Menstrual and treatment history prior to LNG-IUS insertion, including any hormonal or surgical interventions, were documented. Post-insertion outcomes such as changes in haemoglobin levels, duration and severity of bleeding, and side effects were noted. Patient satisfaction and quality of life were assessed using structured questions adapted from the SAMANTA questionnaire telephonically. It is a validated tool designed to detect excessive menstrual blood loss impacting day-to-day functioning. The questionnaire consists of six dichotomous (yes/no) items. Items 1 and 3 are weighted at 3 points each, while Items 2, 4, 5, and 6 are assigned 1 point each, resulting in a total score range of 0 to 10. A score of ≥3 has been validated as the cutoff for identifying heavy menstrual bleeding (HMB) with associated quality-of-life impairment. The tool has demonstrated robust psychometric properties, with sensitivity of 86.7% and specificity of 89.5% in the original validation study [9]. In our study, SAMANTA scores were retrospectively derived from structured follow-up records.

Menstrual blood loss was assessed using the Pictorial Blood Loss Assessment Chart (PBAC), a semi-quantitative tool that estimates menstrual bleeding based on the number and saturation level of sanitary products used, along with the presence of clots and flooding episodes. Each item is assigned a multiplying factor as follows: Lightly stained pad/tampon = 1 point, moderately soiled = 5 points, fully saturated = 20 points, small clot = 1 point, large clot = 5 points, Flooding episode = 20 points. The total score per menstrual cycle is calculated by summing these weighted values. A score >100 per cycle is considered indicative of HMB, based on the original validation by Higham et al. [10].

AUB was defined as any deviation from normal menstrual cycle parameters in terms of frequency, regularity, duration, or volume in reproductive-aged women, as per the FIGO PALM-COEIN classification. HMB was characterised by blood loss that interfered with physical, social, or emotional quality of life and was defined clinically by a PBAC score >100, volume >80 mL or duration exceeding 8 days, based on NICE guidelines. The LNG-IUS used in this study contained 52 mg of levonorgestrel, released at 20 mcg/day. Patient satisfaction was evaluated through structured responses and the SAMANTA questionnaire. The PBAC chart provided a semi-quantitative bleeding assessment based on pad saturation, clot passage, and flooding. The effectiveness of the LNG-IUS was assessed through improvements in haemoglobin levels, reduction in PBAC scores and bleeding/spotting days and overall patient-reported satisfaction.

Statistical analysis was performed using IBM SPSS Statistics for Windows, Version 29 (Released 2024; IBM Corp., Armonk, New York, United States). Descriptive statistics such as mean and standard deviation were calculated. The paired t-test was applied to compare pre- and post-treatment haemoglobin levels and PBAC scores. A p-value < 0.05 was considered statistically significant.

## Results

During the study period, 27 women with AUB who received the levonorgestrel-releasing intrauterine system (LNG-IUS 52 mg) and completed a minimum of six months of follow-up were included. Patient characteristics and clinical profiles are summarised in Table 1.

**Table 1:** Baseline characteristics Data are presented as Mean ± SD. AUB: Abnormal uterine bleeding; PBAC: Pictorial Blood Loss Assessment Chart. A p-value <0.05 was considered statistically significant.

Variable	Number of patients	Mean ± SD
Age (years) Mean (SD)	27	40.3 ± 4.8
Duration of AUB (months)	27	14.6 ± 12.2
Hemoglobin before (g/dL)	27	9.4 ± 1.2
Hemoglobin after (g/dL)	26	11.6 ± 0.72
PBAC score (bleeding)	27	203±64
PBAC pad sub score (per cycle)	27	156.8 ± 53.1
PBAC clot sub score (per cycle)	27	44.74 ± 15.5
PBAC -Flooding sub score	27	0.56 ± 1.5

The mean age of the patients was 40.3 ± 4.8 years (range, 32-48 years), and the majority were multiparous. Comorbidities included hypothyroidism (n=3), hypertension (n=2), and diabetes mellitus (n=1). A history of prior surgery was noted in eight patients (30%). Menstrual patterns prior to LNG-IUS insertion varied, with the majority presenting with heavy menstrual bleeding and prolonged cycles. The mean baseline haemoglobin was 9.4 ± 1.1 g/dL. After LNG-IUS insertion, significant improvements were observed in haemoglobin levels, menstrual flow, and patient-reported symptoms. Post-treatment haemoglobin increased to 11.6 ± 1.2 g/dL (p < 0.0001). The mean PBAC score reduced from 203 ± 26.4 to 78.2 ± 18.5 (p < 0.0001), and the PBAC pad sub score declined significantly (Table 2).

**Table 2:** Pre- and post-treatment comparison of clinical parameters following LNG-IUS insertion * p-value< 0.05 was considered statistically significant. LNG-IUS: Levonorgestrel-Releasing Intrauterine System

Variable	Pre-LNG-IUS	Post-LNG-IUS	p-value
Hemoglobin (g/dL) Mean (SD)	9.4 (1.2)	11.6 (0.72)	<0.0001*
PBAC score	203(64)	78.2 (18.5)	<0.0001*
PBAC pad score	156.8 (53.1)	59.3 (16.4)	<0.0001*
PBAC clot score	44.74 (15.5)	17.2 (6.1)	<0.0001*
Flooding episodes	0.56 (1.5)	0.15 (0.6)	<0.05*
Bleeding > 7 days per cycle	0.74 (0.45)	0.0 (0.0)	<0.0001*
≥3 days of heavy flow	0.93(0.27)	0.19(0.4)	<0.0001*
Uncomfortably abundant periods	0.63(0.49)	0.11(0.32)	<0.0001*
Night-time staining	0.26(0.45)	0.0(0.0)	0.0057*
Worry about seat staining	0.15(0.36)	0.0(0.0)	0.0431*
Avoided activities due to bleeding	0.37(0.49)	0.0(0.0)	0.0006*
Total SAMANTA score	5.81(2.97)	0.52(0.98)	<0.0001*

Based on ultrasound findings and the FIGO PALM-COEIN classification, adenomyosis was the most frequently identified structural abnormality (13 patients, 48%), followed by leiomyomas (five patients, 18%) and endometrial hyperplasia (two patients, 7%). Seven patients (26%) had normal sonographic findings consistent with ovulatory dysfunction.

Side effects reported after LNG-IUS insertion included transient spotting (n=4), pelvic pain (n=2), and breast tenderness (n=1). One patient experienced spontaneous expulsion of the device, and two patients discontinued use, one due to persistent bleeding and other due to weight gain. Amenorrhea was achieved in 10 out of 27 patients (37%) within six months of LNG-IUS insertion and one patient reported amenorrhea by the eighth month. Patients were followed for varying durations ranging from six months to over 24 months. Of the 27 patients, eight (30%) had follow-up between six and 12 months, seven (26%) between 12 and 24 months, and 12 (44%) had more than 24 months of follow-up. Amenorrhea was observed in all groups, with 25% in the shortest group, 43% in the intermediate group, and 42% in the longest group.

Haemoglobin improvement and PBAC score reduction were consistently noted across all follow-up periods, with the most pronounced benefits observed in patients with longer follow-up. Satisfaction remained high throughout, suggesting durable and sustained clinical benefit over time.

Pre-insertion treatment history

Out of 27 patients, 16 (59%) had received prior medical treatment for AUB, such as hormonal therapy. Ten patients (37%) had not received any treatment, and data were unavailable for one. Regarding prior surgical intervention, three had dilation and curettage (D&C), one had D&C with appendicectomy, and one underwent angiography. An additional three patients had unspecified surgical procedures.

Post-insertion outcomes

Amenorrhea was achieved in 10 patients (37%), with the time to amenorrhea ranging from one to eight months (median: six months). Spotting after LNG-IUS insertion was common; 21 patients (78%) experienced prolonged spotting, typically lasting 2-6 months (median: 3 months), while six patients (22%) had no spotting beyond the immediate post-insertion phase. Irregular bleeding was absent in all patients during the initial three months. However, three patients (11%) experienced a recurrence of irregular bleeding after three months, including one who developed irregular cycles two years post-insertion.

Side effects and tolerability

Pelvic pain was reported by four patients (15%), including one with breast tenderness. The remaining 23 patients (85%) reported no pelvic discomfort. Two patients (7%) reported weight gain (~5 kg), while 93% did not. No changes in mood or libido were reported. None of the patients experienced discomfort from LNG-IUS device strings.

Patient satisfaction

Patient satisfaction was high. Twenty-five patients (93%) indicated they would recommend the LNG-IUS to others. Satisfaction was consistent across follow-up durations.

Impact on menstrual bleeding patterns

The use of the LNG-IUS led to marked improvements in menstrual bleeding characteristics. Prior to insertion, 74% of patients reported prolonged bleeding lasting more than seven days per cycle. Following insertion, none of the patients reported bleeding beyond seven days. Similarly, 93% of women experienced at least three days of heavy menstrual flow before treatment, which declined to 19% after LNG-IUS use. The proportion of patients describing their periods as uncomfortably heavy dropped from 63% to just 11% after device insertion.

Issues such as night-time staining of clothes, which affected 26% of patients prior to treatment, were completely resolved post-insertion. Concerns about staining seats during the day also resolved entirely, falling from 15% before insertion to 0% afterward. Furthermore, 37% of patients who previously avoided activities or travel due to frequent pad changes no longer reported such limitations after treatment.

To quantify these improvements in quality of life, the validated SAMANTA questionnaire was applied pre- and post-treatment. The mean total SAMANTA score decreased from 5.81 ± 2.97 to 0.52 ± 0.98 (p < 0.0001), reflecting a 91% reduction in bleeding-related symptom burden. Component-wise scores also declined significantly, as shown in Table 2. These findings collectively reflect a substantial improvement in bleeding control, convenience, and quality of life following LNG-IUS insertion. The overall continuation rate was 93%, with only two patients discontinuing the device within the follow-up period. No serious adverse events, pelvic infections, or pregnancies were reported.

## Discussion

This study demonstrates that the levonorgestrel-releasing intrauterine system (LNG-IUS 52 mg) is highly effective in managing AUB, with significant improvements in haemoglobin levels, reduction in bleeding severity, and high patient satisfaction. The amenorrhea rate of 37% and continuation rate of 93% observed in our study support the therapeutic role of the LNG-IUS as a first-line, uterus-sparing intervention.

Our results are consistent with earlier evidence, including a Cochrane review that demonstrated the superiority of the LNG-IUS over standard medical therapy and comparability with surgical interventions such as endometrial ablation and hysterectomy [11]. More recently, a meta-analysis by Chen et al. reinforced these findings, showing that the LNG-IUS was associated with significantly higher clinical response, better bleeding control and greater patient satisfaction than medical treatments at both six and 12 months [6]. The landmark NEJM randomised trial by Gupta et al. further confirmed these results, reporting that the LNG-IUS was significantly more effective than conventional medical therapy in reducing menstrual blood loss and improving quality of life [12].

The effectiveness of the LNG-IUS appears to vary according to underlying pathology. In our study, patients with endometrial hyperplasia and adenomyosis responded better than those with leiomyoma. This observation mirrors the results of Atak et al., who reported an overall effectiveness rate of 82% but noted marked variation: 95.5% in hyperplasia, 88.7% in adenomyosis, and only 55.6% in leiomyoma cases [8]. Such findings suggest that tailoring expectations and counselling based on etiology may improve patient compliance and satisfaction. These results reinforce the growing evidence that LNG-IUS is a safe, tolerable, and effective alternative to surgical options like hysterectomy, especially in patients who desire uterine preservation or are unfit for surgery. The favourable tolerability profile, high satisfaction, and minimal systemic side effects support its use as a first-line therapy in AUB management protocols. A recent trial by Creinin et al. reported that the LNG-IUS 52 mg achieved more than 90% reduction in menstrual blood loss within six months, further underscoring its efficacy and tolerability [13]. Systematic reviews and narrative analyses, including the review by Bianchi et al., also emphasise its role as a uterus-sparing intervention with consistent findings across diverse healthcare settings [14]. These results are consistent with Indian studies demonstrating high continuation, efficacy, and patient satisfaction with the LNG-IUS in AUB management. Singh et al. reported that the LNG-IUS achieved a marked reduction in menstrual blood loss, significant improvement in haemoglobin levels, and high patient satisfaction, supporting its role as a conservative alternative to hysterectomy in Indian women [15]. A recent institutional study by Shree et al. reported a 48% amenorrhea rate and 88% continuation rate at six months, confirming its acceptability and effectiveness in the Indian setting [16]. Similarly, Dhamangaonkar et al. highlighted the LNG-IUS as an emerging conservative option for women in India, particularly those unwilling or unfit to undergo hysterectomy [17].

Strengths of this study include the use of validated tools (PBAC and SAMANTA), real-world clinical data, and a minimum six-month follow-up. Limitations include the retrospective design, small sample size (n=27), and absence of a control group. PBAC scoring, while semi-quantitative, is subject to patient interpretation and recall bias. Despite these limitations, our results add to the growing body of evidence supporting the LNG-IUS as a safe, tolerable, and highly effective alternative to hysterectomy in women with AUB. Larger, prospective multicenter trials with longer follow-up durations are needed to assess the long-term continuation rate, cost-effectiveness, and impact on fertility or endometrial pathology resolution.

## Conclusions

The LNG-IUS 52 mg demonstrated significant efficacy in this cohort of 27 women with AUB, markedly improving haematological and menstrual parameters reflecting resolution of moderate anaemia and improvement in menstrual bleeding volume and duration. LNG-IUS 52 mg was generally well tolerated with minimal side effects. Transient post-insertion spotting for a few months was common, but other adverse effects were infrequent; no patients reported mood or libido changes. Device retention was excellent, with only one case of spontaneous expulsion and a vast majority (93%) continuing use; only a single patient (3.7%) ultimately required hysterectomy for persistent bleeding.

Patient satisfaction was high, with 93% of participants indicating that they would recommend LNG-IUS 52 mg to others. Overall, the LNG-IUS proved to be a highly effective uterine-preserving therapy for AUB in this study, producing statistically significant reductions in menstrual blood loss and improvements in haemoglobin alongside a favourable safety profile and high acceptability. These findings reinforce the consistent clinical experience of high continuation and patient satisfaction with LNG-IUS in the management of AUB.
